# Fast formation and maturation enhancement of human liver organoids using a liver-organoid-on-a-chip

**DOI:** 10.3389/fcell.2024.1452485

**Published:** 2024-08-14

**Authors:** Jae Hee Byeon, Da Jung Jung, Hyo-Jeong Han, Woo-Chan Son, Gi Seok Jeong

**Affiliations:** ^1^ Biomedical Engineering Research Center, Asan Institute for Life Sciences, Asan Medical Center, Seoul, Republic of Korea; ^2^ Department of Biomedical Sciences, University of Ulsan College of Medicine, Asan Medical Center, Seoul, Republic of Korea; ^3^ Department of Pathology, University of Ulsan College of Medicine, Asan Medical Center, Seoul, Republic of Korea

**Keywords:** microphysiological system (MPS), hepatic models, 3D cell culture, liver microenvironment, liver maturation

## Abstract

**Background:** Spatial and functional hepatic zonation, established by the heterogeneous tissue along the portal–central axis of the liver, is important for ensuring optimal liver function. Researchers have attempted to develop reliable hepatic models to mimic the liver microenvironment and analyze liver function using hepatocytes cultured in the developed systems. However, mimicking the liver microenvironment *in vitro* remains a great challenge owing to the lack of perfusable vascular networks in the model systems and the limitation in maintaining hepatocyte function over time.

**Methods:** In this study, we established a microphysiological system that operated under continuous flush medium flow, thereby allowing the supply of nutrients and oxygen to liver organoids and the removal of waste and release of cytokines therefrom, similar to the function of blood vessels.

**Results:** The application of microphysiological system to organoid culture was advantageous for reducing the differentiation time and enhancing the functional maturity of human liver organoid.

**Conclusion:** Hence, our microphysiological culture system might open the possibility of the miniaturized liver model system into a single device to enable more rational *in vitro* assays of liver response.

## 1 Introduction

Research on the physiology and diseases of the human liver have been limited owing to the difficulty of maintaining sustainable *in vitro* organ cultures that take into consideration the liver microenvironment. Most of the conventional *in vitro* liver models have been unable to fully replicate the complex tissue architecture, regenerative capacity, and functions of this organ. Under these limited conditions, two-dimensional (2D) mono-layered cells exhibit altered biological features, including morphology, function, hepatic polarity, and genotype expression (Lasser et al., 2002; Lee, 2003; Ma et al., 2023). To overcome the limitations of these 2D *in vitro* models, three-dimensional (3D) cell cultures—especially organoids—have been proposed for mimicking the architecture and genetic expression of native human liver tissue (Akbari et al., 2019; Sorrentino et al., 2020).

In recent years, researchers have focused on developing primary tissue-derived liver organoids in a 3D cellular architecture, thereby attempting to recapitulate the characteristics of native liver tissue (Huch et al., 2015; Broutier et al., 2017). The liver organoid has self-renewal capability, a key proliferative property for its long-term culture and clonal expansion from single cells with sustained stemness (Huch et al., 2013; Hu et al., 2018). Furthermore, the organoid could recapitulate several liver diseases, including fatty liver disease, cirrhosis, and monogenic metabolic diseases (Guan et al., 2017; Wang et al., 2020; Guan et al., 2021). Results have shown liver organoids to be a promising cell source for *in vitro* studies on the developmental biology and pathophysiology of the liver and hepatic system.

Researchers have used primary human liver tissue, hepatic bipotent stem cells, and hepatocytes to establish organoids that can undergo self-organized tissue formation (Huch et al., 2015; Hu et al., 2018). These cultured organoids can then be differentiated into liver cells with various characteristics. However, it has been challenging to maintain long-term cultures and develop the phenotype and functions of fully mature hepatocytes. In particular, the conventional droplet-based culture conditions do not possess the unique hepatic microenvironment required to preserve biochemical gradients, such as those of nutrients, oxygen tension, and metabolites. In terms of its anatomical structure, the liver is organized into units known as lobules (Trefts et al., 2017). Blood flow from the periportal to the perivenous zones along the lobular axis creates spatial gradients of oxygen, metabolites, and nutrients, which benefit hepatocytes and other cells (Tsukada et al., 2013; Ben-Moshe and Itzkovitz, 2019). The liver responses to these gradients are likely to contribute to the development of differences in cellular contexts between hepatic cells. Therefore, a culture system that can overcome these defects is still needed.

Physiological relevant *in vitro* models provide a promising platform for modelling the physiochemical conditions of the hepatic microenvironment (Janani and Mandal, 2021; Kostrzewski et al., 2021; Paradiso et al., 2023). The preliminary experimental works toward the development of hepatic cultures *in vitro* have focused on cultivates cells in a 3D system composed of an extracellular matrix (ECM) (Farhan et al., 2023; Zhu et al., 2023). The Integration of the perfusion channel for dynamic culture has allowed the recapitulation of *in vivo* conditions, leading to spatiotemporal distribution of oxygen, nutrients and metabolic wastes (Yu et al., 2017; Lekkala et al., 2024). Although recent advances in the microphysiological systems (MPSs) capacity, but there remain hurdles to overcome. These challenges include complex procedure requiring specialized equipment and materials, as well as an absence of organoid size control (Balakrishnan et al., 2015; Schuster et al., 2020).

For this study, we hypothesized that an *in vivo*-like biochemical gradient would help to maintain the physiological microenvironment of the ductal liver tissue and enhance the maturation and differentiation of the human liver organoid (hLO). Our microfluidic platform provides an easy-to-use, low-cost, and single-step 3D microfluidic device for culturing human liver organoids. Moreover, this system is designed to localize organoids at approximately the same position along the fabricated microfluidic channel, so organoids can be exposed to similar environmental conditions. The self-assembled hLOs cultured in the MPS displayed phenotypic and functional enhancements over those formed using the conventional droplet culture method, indicating that the MPS had additional beneficial effects on the maturity of the organoids as it provided a more *in-vivo*-like cellular microenvironment.

## 2 Materials and methods

### 2.1 Liver sample

The adjacent non-tumor liver tissue from a consented patient who underwent surgical resection at the Asan Medical Center (Seoul, South Korea) was obtained.

### 2.2 Generation of the human liver organoid-integrated microphysiological system

**FIGURE 1:**
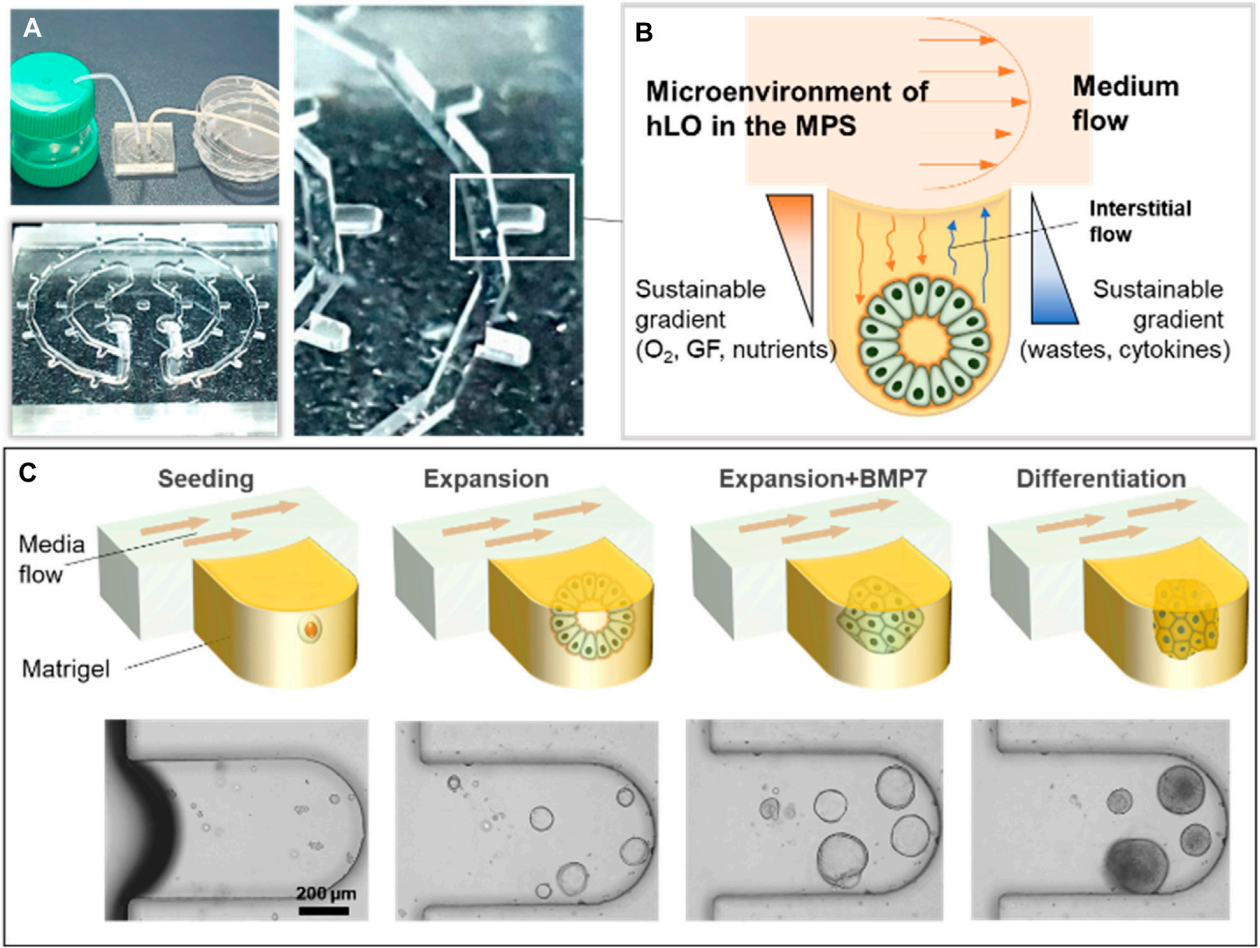
Schematic diagram of the microphysiological system for culturing human liver organoids. **(A)** Photographs showing examples of the experimental set-up for the MPS. **(B)** Illustration of the operating principle of hLO formation using the MPS device. **(C)** The mixture of mechanically dissociated cells was seeded in the MPS and cultured in the expansion medium for 4 days. Then, the cells were treated with BMP7 for an additional 2 days. The medium was changed to the differentiation medium for 5 days. MP,: microphysiological system; GF, growth factors; hLO, human liver organoid; BMP7, bone morphogenetic protein 7.

To generate the hLO-integrated MPS, a microfluidic device was fabricated as previously described (Jung et al., 2019; Jung et al., 2020). In brief, the poly (dimethylsiloxane) (PDMS; Sylgard^®^ 184; Dow Chemical Co., Midland, MI, United States) microchannel was replicated from a patterned SU-8 master mold, which was produced on a silicon wafer using conventional soft lithography (MicroChem, Newton, MA, United States). A mixture of a PDMS pre-polymer containing a PDMS precursor and a curing agent at a 10:1 ratio was decanted into the SU-8 mold and solidified in a drying oven (at 60°C) for 2 h. Inlet and outlet holes were then created using a hole puncher (diameter, 2 mm). The PDMS channel and cover glass were then sterilized in an autoclave (JEIO TECH, Daejeon, Korea), following which the channel was bonded to the cover glass after treatment with oxygen plasma (Femto Science, Hwaseong, Korea) (Figure 1A). Isolated the liver ductal cells were embedded in Matrigel and selective isolation of hepatic bipotent stem cells was achieved using isolation medium until organoids were visible. Organoids were subsequently dissociated and seeded with 50% (v/v) growth factor-reduced Matrigel into the microfluidic device via centrifugal force (Supplementary Figure S1). The remaining cells and Matrigel in the channel were washed out, and the Matrigel in the microwells was solidified at 37°C for 30 min. Using Teflon tubes, the microfluidic device was connected to the inlet and drain reservoir to allow the continuous supply of hLO expansion medium. A yarn flow resistor was used to control the flow rate of each culture medium (Figure 1A).

### 2.3 Demonstration of concentration gradients in the MPS

Matrigel was diluted in media (50% v/v) was introduced to the chip through the inlet of the device using centrifugal force (microcentrifuge) at a maximum speed within a few seconds and remaining Matrigel in the channel were removed. The medium reservoir containing 5 μM FITC (46950, Sigma–Aldrich) was filled with PBS. PBS with FITC was pumped through the medium inlet to establish a vertical FITC concentration gradient from the medium channel to the bottom of the microwells. Fluorescent images were acquired every 20 min using EVOS FL Auto 2 microscope (Thermo Fisher Scientific). The sequential timelapse fluorescent images were converted to movie files using Clipchamp.

### 2.4 Human hepatic bipotent stem cell culture

Human liver tissue was collected and finely minced in HBSS (14175-095, Gibco, Waltham, MA, United States). Using a pipette aid, the tissue was mechanically disrupted by pipetting it up and down several times, after which it was allowed to settle, and the supernatant was aspirated to remove fat and debris. To isolate the liver ductal cells, the fine tissue was incubated in a human liver digestion solution containing 2.5 mg/mL collagenase D from *Clostridium histolyticum* (11088882001, Roche, Basel, Switzerland) and 0.1 mg/mL DNase I (DN25, Sigma-Aldrich, St. Louis, MO, United States) in HBSS for 45 min at 37°C. The cells were then filtered with a 40 μm strainer (63040, SPL Life Sciences, Pocheon, Korea) and washed with basal medium for 5 min at 8°C. The basal medium contained 1% penicillin–streptomycin, 1% GlutaMAX (35050061, Gibco), and 10 mM HEPES (15630, Gibco) in Advanced DMEM/F-12 (12634, Gibco). To generate the hLOs, bipotent stem cells (>50,000) were embedded in growth factor-reduced Matrigel (D356231, Corning Inc., Corning, NY, United States) and seeded in 24-well plates. Cells were cultured in hLO isolation medium containing 25 ng/mL recombinant human Noggin (120-10C, PeproTech, Rocky Hill, NJ, United States), 30% (vol/vol) Wnt3a-conditioned medium, 10% (vol/vol) R-Spondin 1 (Rspo1)-conditioned medium, and 10 µM of the Rho kinase (ROCK) inhibitor Y-27632 dihydrochloride (1254, Tocris Bioscience, Bristol, United Kingdom) as well as 1:50 B27 supplement without vitamin A (12587010, Life Technologies, Carlsbad, CA, United States), 1:100 N2 supplement (17502-048, Life Technologies), 1 mM *N*-acetylcysteine (5619, Tocris Bioscience), 10 mM nicotinamide (N0636, Sigma-Aldrich), 10 nM recombinant human [Leu^15^]-gastrin I (G9145, Sigma-Aldrich), 50 ng/mL recombinant human epidermal growth factor (EGF; AF-100-15, Peprotech), 100 ng/mL recombinant human fibroblast growth factor (FGF) 10 (100-26, PeproTech), 25 ng/mL recombinant human hepatocyte growth factor (HGF; 100-39, Peprotech), 10 µM Forskolin (1099, Tocris Bioscience), and 5 µM of the transforming growth factor-beta (TGFβ) inhibitor A83-01 (2939, Tocris Bioscience) in basal medium until the appropriate organoid formation. After generation of the organoids, the medium was changed to an hLO expansion medium containing 1:50 B27 supplement without vitamin A, 1:100 N2 supplement, 1 mM *N*-acetylcysteine, 10% (vol/vol) Rspo1-conditioned medium, 10 mM nicotinamide, 10 nM recombinant human [Leu^15^]-gastrin I, 50 ng/mL recombinant human EGF, 100 ng/mL recombinant human FGF10, 25 ng/mL recombinant human HGF, 10 µM Forskolin, and 5 µM A83-01 in basal medium. For the Matrigel droplet cultures in a static format, organoids were cultured for 6 days. For the MPS cultures, organoids were cultured for 4 days. Before differentiation of the hLOs to hepatocytes, 25 ng/mL bone morphogenetic protein 7 (BMP7; 120-03P, PeproTech) was added to the Matrigel droplet culture or MPS culture, and the cells were cultured for an additional 5 days and 2 days, respectively. The hLOs from the Matrigel droplet and MPS had respectively been cultured 12 or 5 days in a differentiation medium comprising basal medium supplemented with 1:50 vitamin A-containing B27 supplement, 1:100 N2 supplement, 1 mM *N*-acetylcysteine, 10 nM recombinant human [Leu^15^]-gastrin I, 50 ng/mL recombinant human EGF, 25 ng/mL recombinant human HGF, 0.5 µM A83-01, 10 µM DAPT, 3 µM dexamethasone (D1756, Sigma-Aldrich), 25 ng/mL BMP7, and 100 ng/mL recombinant human FGF19 (100-32, PeproTech).

### 2.5 Quantitative reverse transcription-polymerase chain reaction

At the endpoint of experiments, organoids were removed from Matrigel, digested with TrypLE (12605010, Gibco™), washed with PBS. Total RNA was isolated using mirVANA miRNA isolation kit (AM1561, Ambion, Austin, TX, United States) according to the manufacturer’s extraction protocol used to isolate total RNA. cDNA was synthesized from 0.18 µg of RNA using the PrimeScript™ First Strand cDNA Synthesis Kit (6110 A, Takara, Seoul, Korea). A Roche LightCycler 480 instrument and a SYBR Green I Master kit were used to amplify the target genes. The relative expression level of each gene, which was normalized to that of glyceraldehyde 3-phosphate dehydrogenase (*GAPDH*), was calculated using the 2^−ΔΔCT^ method. Data were expressed as mean ± S.D. of triplicate experiments. Supplementary Table S1 shows the primer sequences used for detecting the target genes.

### 2.6 Flow cytometry

Cells were fixed and stained using Cytofix/Cytoperm Kit (554714, BD) following the manufacturer’s instructions. For staining of intracellular A1AT, dissociated cells were pelleted by centrifugation and resuspended in Fixation/Permeabilization solution from Cytofix/Cytoperm Kit on ice for 20 min. Cells were then washed twice with 1X Perm/Wash™ Buffer. After washing, cells were incubated with the primary antibody listed in Supplementary Table S2 for 30 min A1AT expression was determined based on samples stained with the isotype controls. Cells were analyzed using a BD FACSAria II flow cytometer (BD Biosciences).

### 2.7 Immunofluorescence assay

The hLOs were embedded in a HistoGel block (HG-4000-012, Thermo Fisher Scientific, Waltham, MA, United States) and further embedded in paraffin. Thereafter, the paraffin block was sliced into 7 μm sections, which were placed on slides, de-paraffinized with xylene, and re-hydrated with ethanol. After antigen retrieval, the slides were incubated with H_2_O_2_ for 10 min to reduce the background signal. Non-specific epitopes were then blocked by incubating the sections with 5% goat serum for 1 h. Thereafter, the sections were incubated overnight with the primary antibody at 4°C, washed with TBST solution, and subsequently incubated with the secondary antibody (1:1,000 to PBS) at room temperature for 2 h. After washing off the secondary antibody with TBST, DAPI solution was added for nuclear staining of the cells. Finally, the sections were washed with TBST, covered with a glass cover using mounting solution, and observed under a confocal microscope (LSM 780, Carl Zeiss, Jena, Germany). Supplementary Table S2 lists the primary antibodies used in this study.

### 2.8 Tissue clearing

Before whole-mount staining of the hLOs, a clearing step was carried out. In brief, the fixed organoids were embedded in hydrogel (C1310X X-Clarity Hydrogel Solution Kit, Logos Biosystems, Inc., Anyang, Korea) and incubated at 4°C for 1 day. After further incubation at 37°C for 3 h to allow polymerization, a tissue-clearing solution (C13001, Electrophoretic Tissue Clearing Solution, Logos Biosystems, Inc.) was added to the hLOs for 2 days. The organoid became transparent after clearing, they were then processed for immunofluorescence staining.

### 2.9 Enzyme-linked immunoassay of albumin

To quantify the albumin levels in the culture media, culture supernatants were collected and centrifuged at 1,000 ×g for 5 min to remove debris. The final supernatants were frozen (−80°C) until use. Due to the culture volume being larger than it was for the Matrigel droplet culture under static conditions, the cell supernatants from the MPS were concentrated using Amicon Ultra centrifugal filter devices (Millipore) before ELISAs were performed. The secreted albumin was then analyzed using a Human Albumin AssayMax™ ELISA kit (EA3201-1, Assaypro, St. Charles, MI, United States) according to the manufacturer’s protocol. The absorbance at 450 nm wavelength was recorded using a microplate reader.

### 2.10 Assay of metabolites

#### 2.10.1 Materials

All standard metabolites and internal standards were purchased from Sigma-Aldrich. All solvents, including water, were purchased from J. T. Baker (Phillipsburg, NJ, United States).

#### 2.10.2 Sample preparation

In brief, 30 μL of cell medium was mixed with 500 μL of cold acetonitrile and 30 μL of internal standard solution (1 μM cholic acid-d5 solution), and the solution was centrifuged at 13,000 rpm and 4°C for 10 min. The supernatant was collected and dried using a vacuum centrifuge and stored at −20°C until LC-MS/MS analysis. The dried sample was reconstituted with 50% methanol before injection into the LC-MS/MS system.

#### 2.10.3 Liquid chromatography-tandem mass spectrometry

The lipid levels of bile acids were determined using an LC-MS/MS system equipped with a 1290 HPLC system (Agilent, Waldbronn, Germany) and QTRAP 5500 mass spectrometer (AB Sciex, Toronto, ON, Canada). A reverse-phase column (Pursuit 5 C18, 150 × 2.1 mm, Agilent) was used with mobile phase A (7.5 mM ammonium acetate, set to pH 4 using 10 M acetic acid) and mobile phase B (5% acetonitrile in methanol). The LC was run at a flow rate of 200 μL/min at 24°C with the following mobile phase gradient: 40% of A for 0 min, 40%–20% of A for 20 min, 20%–10% of A for 5 min, 10% of A for 5 min, 10%–40% of A for 5.1 min, and 40% of A for 4.9 min. Multiple reaction monitoring was performed in the negative ion mode, and the extracted ion chromatogram corresponding to the specific transition for each bile acid was used to quantify the lipid levels. The calibration range for each lipid was 0.1–10,000 nM (*r* ^2^ ≥ 0.99). Data analysis was performed using Analyst 1.5.2.

### 2.11 Statistical analysis

Statistical analysis of all data was performed using the unpaired Student’s t-test with Prism 8 software (GraphPad, Inc., San Diego, CA, United States). A *P*-value of less than 0.05 between groups was considered statistically significant.

## 3 Results

### 3.1 Culturing of human liver organoids in the microphysiological system

To culture hLOs under dynamic medium flow conditions, a medium flow controller was applied during the expansion and differentiation of the organoids. The expansion and differentiation culture media were siphoned from the medium reservoir, passed through the 29 microwells of the device in which organoids were forming and expanding, and then drained into a drain reservoir (Figure 1A). Hence, through the process of diffusion, the soluble factors, growth factors, nutrients, and oxygen were indirectly supplied to the hLOs in the microwells. In parallel, metabolic waste and cytokines released from the hLOs were also diffused into the media and washed out through the medium flow (Figure 1B; Supplementary Movies S1, S2). In this study, the entire process of hLO formation from seeding to differentiation was performed in the MPS (Figure 1C).

### 3.2 Comparison of the expansion and differentiation of human liver organoids in droplet and dynamic flow cultures

**FIGURE 2:**
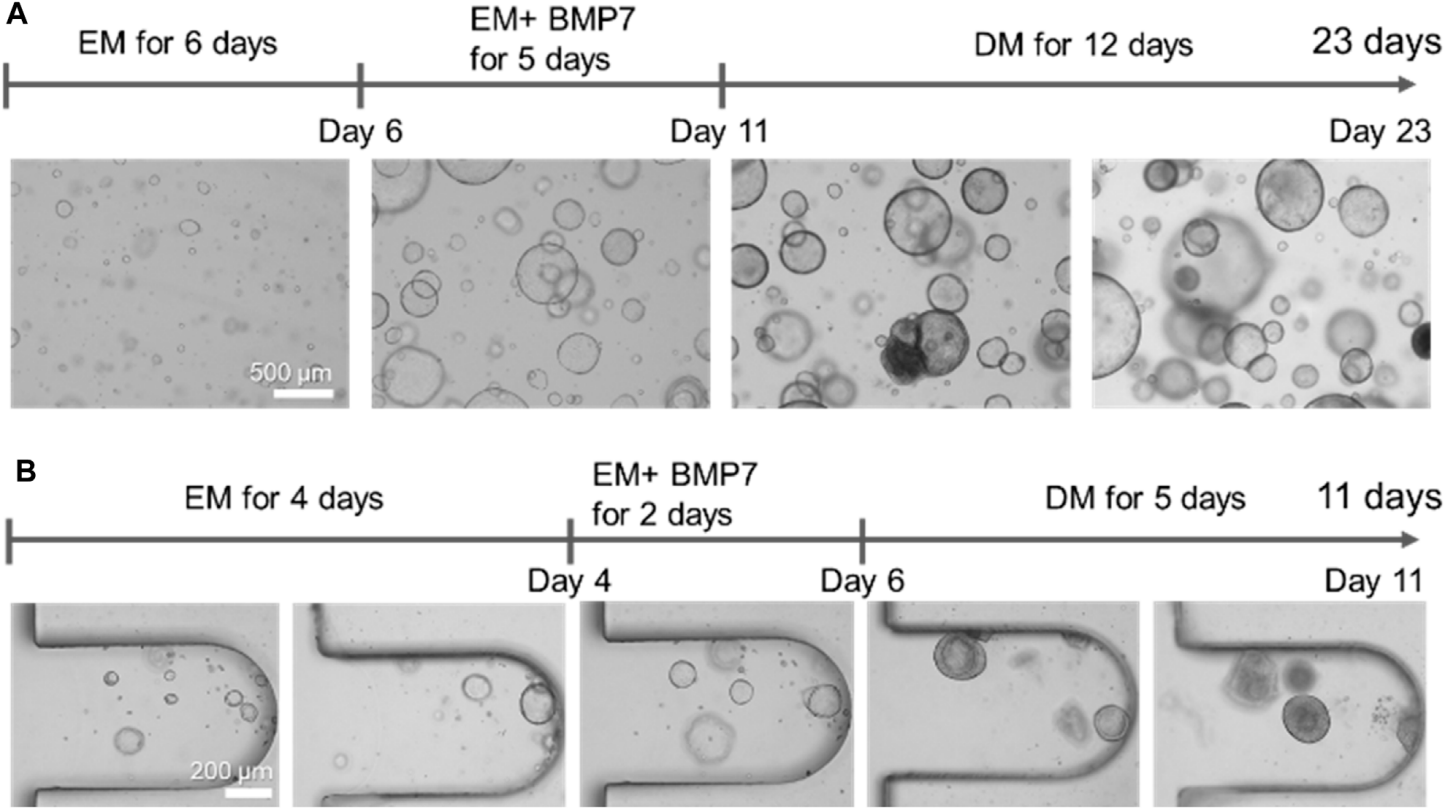
Comparison of the morphologies of human liver organoids cultured in Matrigel droplets or the microphysiological system. **(A)** Morphologies of the organoids formed on Matrigel droplets at the indicated time points. Bar, 500 μm. **(B)** Morphologies of the organoids formed on the microfluidic-based organoid culture device at the indicated time points. Bar, 200 μm. EM, expansion medium; BMP7, bone morphogenetic protein 7; DM, differentiation medium.

The organoids were embedded in a Matrigel droplet or the MPS to compare the hLOs formed under conventional and dynamic flow culture conditions. In brief, hLOs were cultured in the isolation medium and then mechanically disrupted using a pipette. The majority of the organoids were less than 50 µm in size. After the disruption process, the hLO fragments were seeded in a Matrigel droplet or the MPS. The fragments that formed a budding cyst-like self-assembled organoid could be enlarged by culturing in the expansion medium for 6 days. BMP7 is required as an additional medium component before starting hLO differentiation into hepatocytes. During the expansion process, the hLOs in the Matrigel droplet were observed to be varied in size, with those near the boundary of the droplet being larger than those at the core region (Supplementary Figure S2). The induction of hLO differentiation into hepatocyte-like cells was then initiated by the addition of DAPT and dexamethasone, which are required to promote hepatic differentiation for 12 days or longer (Figure 2A). Morphological heterogeneity was observed in some hLOs in the droplet, and these changes occurred irregularly during differentiation. Regarding the duration for differentiation, hLOs embedded in the Matrigel droplet typically needed a minimum of 21 days or longer to fully differentiate. In contrast, the hLOs in the MPS were cultured in the expansion and differentiation media for 5 and 6 days, respectively, with the complete expansion and differentiation processes requiring a total time of approximately 11 days only (Figure 2B). During expansion, the hLO formed a spherical 3D cystic structure in a single layer in the microwell. Interestingly, Matrigel droplet cultures often suffer from heterogeneous seeding effects and growth (Figure 2A; Supplementary Figure S2). In contrast, oxygen and nutrient diffusion is less limited in MPS due to its relatively thin layer of Matrigel, resulting in a narrow range of variation in the size and morphological heterogeneity of the organoids (Figure 2B; Supplementary Figure S3). The structure gradually became compacted and condensed during the differentiation process. These results showed that continuous medium flow in an MPS could accelerate hepatic differentiation by creating a favorable microenvironment that supports the formation of well-defined and homogeneous structured hLOs.

### 3.3 Characterization of liver organoids maintained in the expansion medium

**FIGURE 3:**
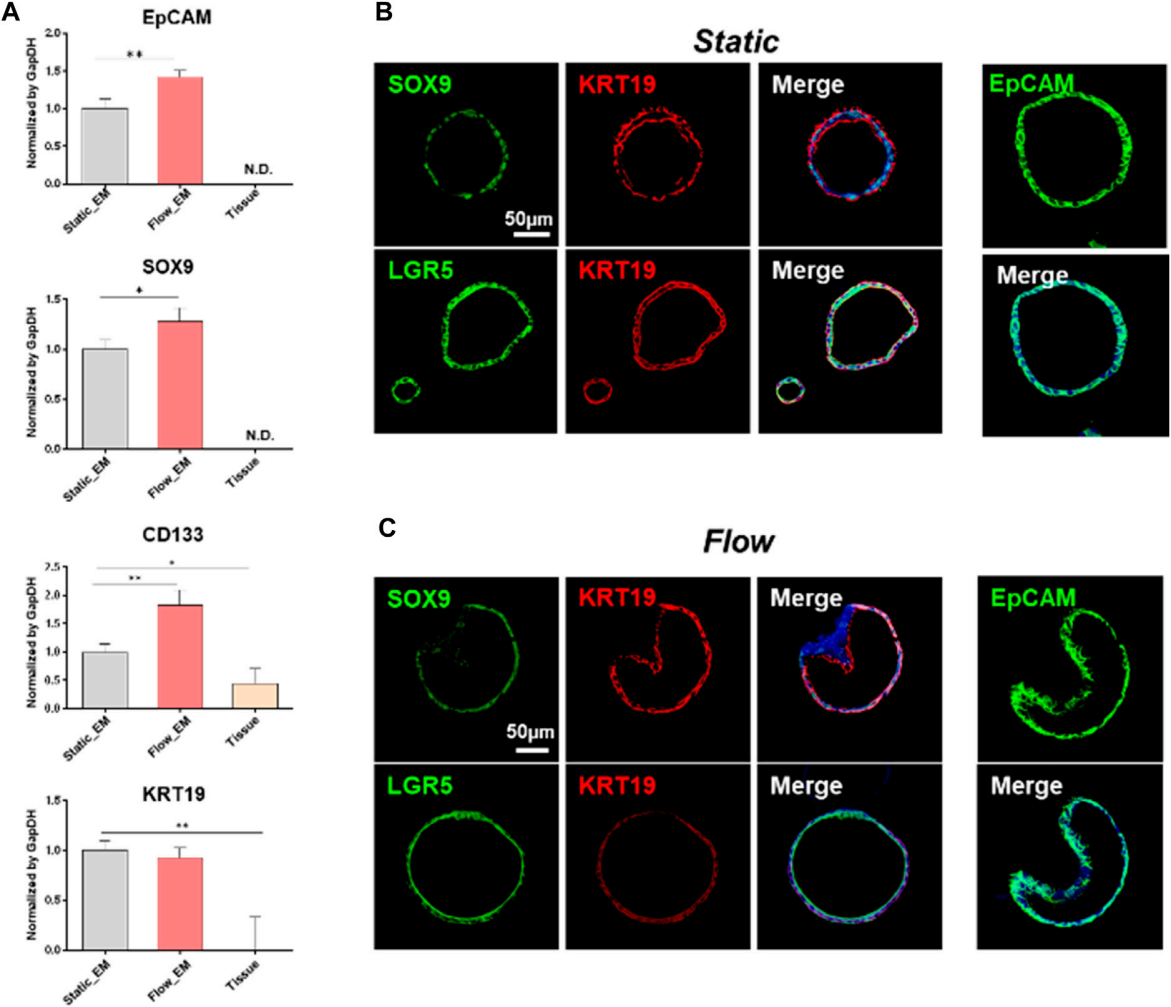
Characterisation of liver tissue-derived organoids maintained in an expansion medium. **(A)** mRNA expression of key liver progenitor/bipotent markers (*EpCAM*, *SOX9*, *CD133*, and *KRT19*), as analyzed by qRT-PCR on the last day of expansion. The gene expression levels of the tissue-derived organoids in static or flow cultures were compared with those of human liver tissue. Error bars show the means ± S.D. of biological triplicates. **(B, C)** Immunofluorescence staining for progenitor/bipotent markers (EpCAM, KRT19, LGR5, and SOX9) in static **(B)** and flow **(C)** cultures. The scale bars in **(B)** and **(C)** represent 50 μm. EpCAM, epithelial cell adhesion molecule; SOX9, SRY-box transcription factor 9; CD133, prominin 1; KRT19, type 1 cytoskeletal 19 (or keratin 19); LGR5, leucine-rich repeat-containing G-protein coupled receptor 5.

To characterize the liver organoids maintained in the expansion medium, qRT-PCR (Figure 3A) and immunofluorescence staining analyses were performed (Figures 3B, C) on the last day of organoid expansion. The organoids derived from adult liver ductal cells expressed liver progenitor/bipotent markers and ductal cell markers. The genes coding for epithelial cell adhesion molecule (*EpCAM*, known to take part in the formation of liver organoids) and SRY-box transcription factor 9 (*SOX9*, a liver progenitor marker) were expressed under static and flow conditions but not in the liver tissue. The expression level of the prominin 1-encoding gene (*CD133*, a liver oval cell/hepatoblast marker) was higher in the hLOs from the MPS than in those from the conventional droplet culture. The gene coding for type 1 cytoskeletal 19 (*KRT19*, also known as keratin 19, a ductal cell marker) was expressed at similar levels under static and flow conditions. These findings were further confirmed at the protein level, at which there were no significant differences between the flow and static culture systems regarding immunofluorescence staining for the stem/progenitor markers SOX9, LGR5, KRT19, and EpCAM.

### 3.4 Expression of albumin after hLO differentiation

**FIGURE 4:**
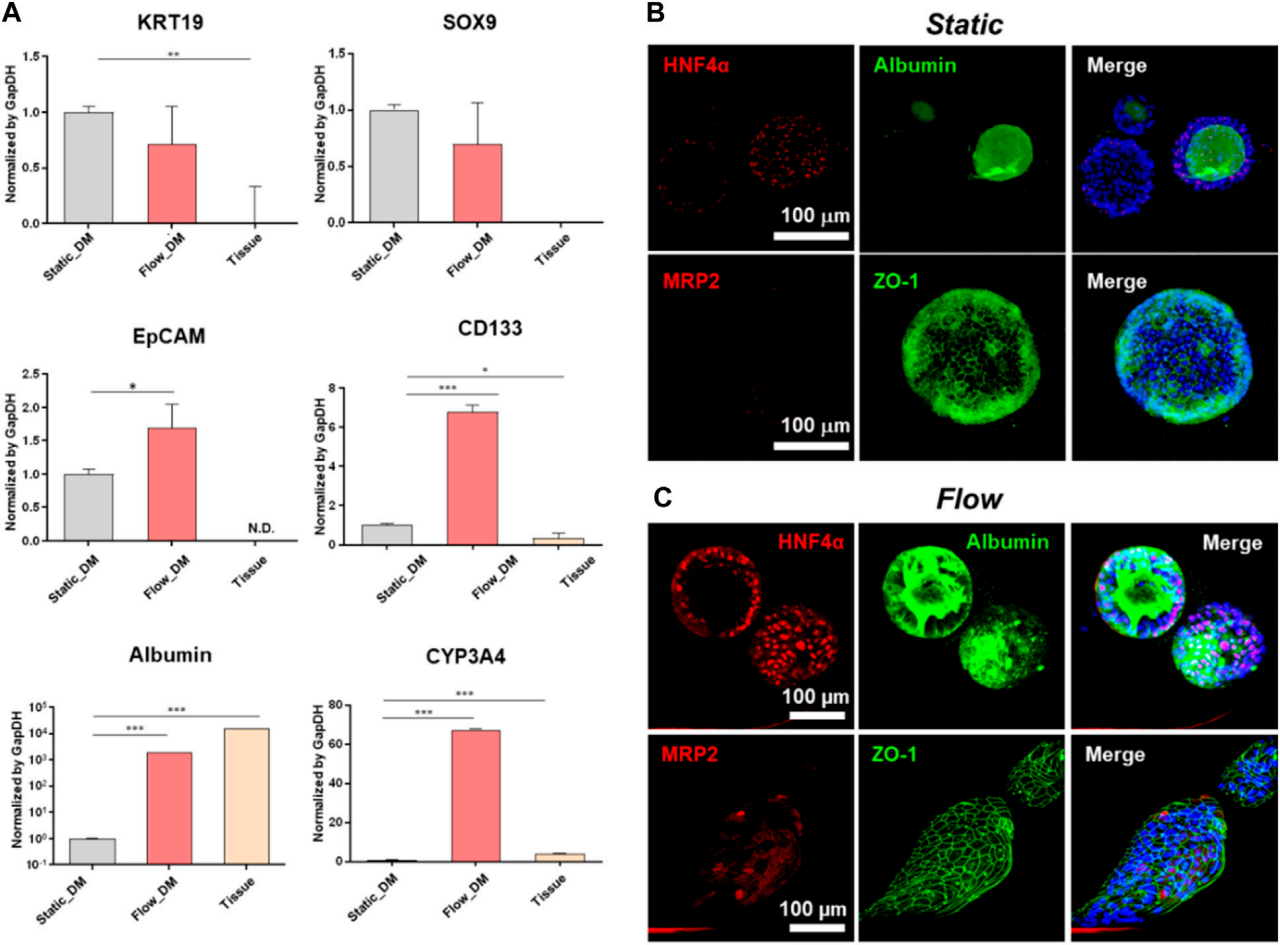
Characterization of the differentiation of tissue-derived organoids to hepatocytes. **(A)** mRNA expression of progenitor and hepatocyte markers (*EpCAM*, *SOX9*, *CD133*, *KRT19*, albumin, and *CYP3A4*) analyzed by qRT-PCR on the last day of differentiation. The expression levels of these genes in the tissue-derived organoids in the static or flow cultures were compared with those of human liver tissue. The error bars show the means ± S.D. of biological triplicates. Whole-mount immunofluorescence staining of MRP2, albumin, ZO-1, and HNF4α following differentiation under either static **(B)** or flow conditions **(C)**, followed by tissue clearing. The scale bars in **(B, C)** represent 100 μm. EpCAM, epithelial cell adhesion molecule; SOX9, SRY-box transcription factor 9; CD133, prominin 1; KRT19, type 1 cytoskeletal 19 (or keratin 19); CYP3A4, cytochrome P450 family 3 subfamily A member 4; MRP2, multidrug resistance-associated protein 2; ZO-1, zona occludens protein 1; HNF4α, hepatocyte nuclear factor 4-alpha.

hLO differentiation to hepatocyte-like cells is accomplished by changing the culture medium to one supplemented with DAPT and dexamethasone but without Rspo1. We analyzed the RNA and protein expression of several markers after 12 days of culture under the static condition or 5 days under the flow condition. The level of differentiation was investigated by qRT-PCR assay using the hepatocyte markers albumin and *CYP3A4* (Figure 4A). Both markers were highly expressed under the flow condition compared with under the static condition. Moreover, we performed whole-mount organoid staining with a clearing step (Figures 4B, C). Polygonal membrane networks with positive staining for zona occludens protein 1 (ZO-1), and the hepatocyte markers hepatocyte nuclear factor 4-alpha (HNF4α), multidrug resistance-associated protein 2 (MRP2), and albumin were detected in both the static and flow systems. However, MRP2 expression under static was scarcer than those found in MPS. In addition, the polygonal structure and expression of hepatocyte markers were more clearly evident under the flow condition (Figure 4C). In particular, albumin was highly accumulated inside the organoids formed in the fluidic system (Figure 4C).

### 3.5 Functional analysis of the hepatocyte-like cells

**FIGURE 5:**
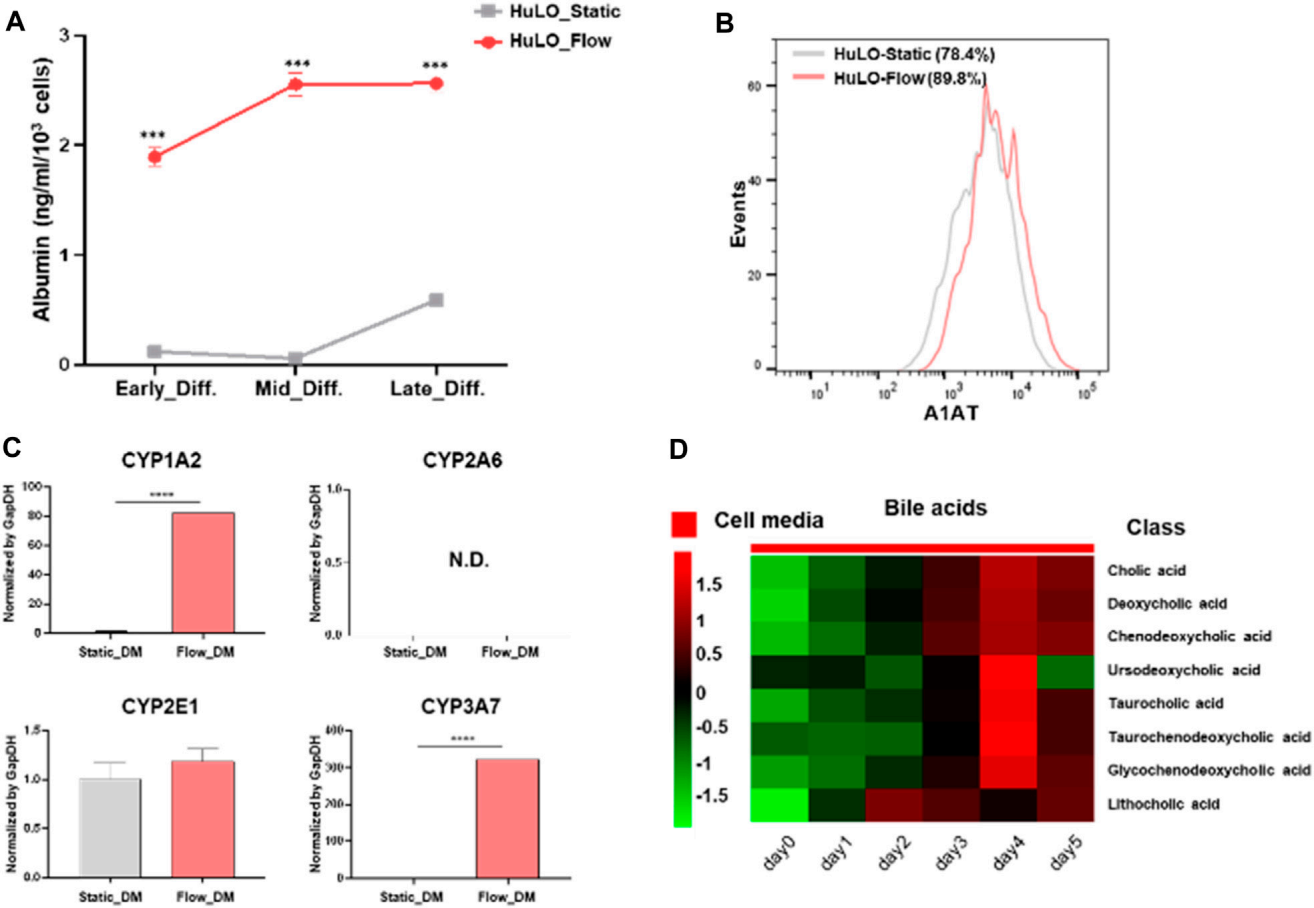
Functional characterization of hepatocyte-like cells differentiated from tissue-derived organoids. **(A)** Albumin concentrations in the supernatant were measured by ELISA (ng/ml/10^3^ cells) on the last day of differentiation. The data are expressed as the mean ± S.D. from triplicate experiments. **(B)** Representative histograms of the A1AT expression after hepatic differentiation of organoids under the static (grey) or flow (pink). **(C)** Relative basal expression of CYP1A2, 2A6, 2E1, and 3A7 in hLOs shown as genes among samples. The unpaired Student’s t-test was used to determine the statistical significance of the data (****P < 0.001). **(D)** Heat map of various bile acid classes. EM, expansion medium; DM, differentiation medium.

To verify that hepatic differentiation had been achieved with our MPS, the functions of the hepatocyte-like cells were evaluated. The culture supernatants were collected on the last day of incubation, and ELISA was performed for quantitative measurement of the albumin content. Albumin is present at higher levels in the fluidic system compared with the static condition (Figure 5A). Additionally, we characterized the additional markers of functional hepatocytes, including α1-antitrypsin (A1AT) and cytochrome P450 (CYP450) isoforms. As expected, the A1AT expression level in MPS after differentiation increased by approximately 10% than the static condition (Figure 5B). To further delineate the maturity of hLO under flow condition, we assessed the presence of hepatocyte specification marker, asialoglycoprotein receptor 1 (ASGPR1), an endocytotic cell surface receptor specific to adult hepatocytes (Supplementary Figure S4). The results presented in Figure 5C indicate that several P450 enzymes expressed higher in fluidic systems than static. Expression of CYP2A6 could not be detected in any of the samples analyzed. Additionally, cultured media were collected every day after differentiation to analyze metabolites. As shown in Figure 5D, bile acid-related metabolites were secreted in high amounts on day 4. We could presume that CYP3A4 was activated by endogenous bile acid for detoxification of the latter. Overall, hepatocytes produced under the dynamic medium flow condition functionally outperformed those from the conventional droplet culture in albumin production, upregulation of CYP 450s expression, and accumulation of bile acids.

## 4 Discussion

The liver is a spatially and functionally heterogeneous organ that contains branches of the hepatic portal vein, hepatic artery, and biliary tree across its lobules (Ben-Moshe et al., 2019; Inverso et al., 2021). The spatial heterogeneity in the liver serves a wide variety of physiological functions, including xenobiotic metabolism, protein and bile synthesis, and the dynamics of hepatic regeneration (Li and Chiang, 2014; Tanami et al., 2017). The liver could be a useful tool in drug development and regenerative medicine, from early-stage drug discovery to preclinical trials. However, many current *in vitro* liver models are limited by their lack of proper hepatic functions, the loss of the hepatic microenvironment, and the short lifespan of the hepatic cells in culture.

Recently, stem cell-based 3D culture methods have been developed for the formation of organoids (Sato et al., 2011; Fatehullah et al., 2016; Boonekamp et al., 2019). These self-organizing 3D structures are formed by embedding tissue-resident stem cells in Matrigel and then culturing them in media supplemented with growth factors based on the endogenous stem cell niche (Broutier et al., 2016). Self-renewing liver organoids demonstrating genetic stability during long-term culture were reported in 2015 (Huch et al., 2015). According to previous reports, isolated healthy ductal cells (or *LGR5*-positive liver cells post-damage induction) could self-organize into 3D structures that sustained long-term expansion as adult ductal progenitor cells while retaining their ability to differentiate into functional hepatocyte-like cells *in vitro*.

However, the Matrigel-based droplet culture cannot provide nutrients and oxygen exchange and limits the amount of metabolic waste being removed from the interior of the organoids. Owing to the limited diffusion of oxygen and nutrients into their core, organoids of irregular size were generated, and differences in the expansion and differentiation stages occurred. Depending on the hepatic cell position in the hepatic lobule and the corresponding differences in exposure to oxygen and nutrient gradients, heterogeneity and functional plasticity arose among the hepatic cells (Tonon et al., 2019). Therefore, the provision of controlled oxygen and nutrient gradients to create functional hepatocyte regional variations similar to those observed *in vivo* is required.

Herein, we have presented an MPS in which nutrients are supplied, and waste is removed *via* continuous flush medium flow, similar to that *in vivo*. hLOs cultured in the MPS presented as self-assembled organoids, and the maturation period of the hepatic cells was shorter than that in static Matrigel droplet culture. Additionally, implementation of the *in vivo*-like biochemical gradient in the MPS influenced the expression of liver-specific genes and improved the hepatocyte-like cell biological properties, including albumin production, upregulation of CYP 450s expression, and accumulation of bile acids. Thus, this MPS provides an environment that supports the maturity of hLOs as it recapitulates a more *in vivo*-like cellular microenvironment.

## Data Availability

The datasets presented in this study can be found in online repositories. The names of the repository/repositories and accession number(s) can be found in the article/Supplementary Material.
