# Supplementary material for: Fast formation and maturation enhancement of human liver organoids using a liver-organoid-on-a-chip
Source: Front Cell Dev Biol. 2024 Aug 14;12:1452485. doi: 10.3389/fcell.2024.1452485 (PMC11349704; doi:10.3389/fcell.2024.1452485)
Supplement: Supplementary file 1 [file DataSheet1.pdf]

Supplementary materials

Table S1 Primer sequences used for the qRT-PCR.

Albumin	F: ATGCCCCGGAACTCCTTTTC
	R: CAACAGGCAGGCAGCTTTAT
CD133	F: GGACCCATTGGCATTCTC
	R: CAGGACACAGCATAGAATAATC
CYP1A2	F: CTTCGCTACCTGCCTAACCC
	R: GACTGTGTCAAATCCTGCTCC
CYP2A6	F: CAGCACTTCCTGAATGAG
	R: AGGTGACTGGGAGGACTTGAGGC
CYP2E1	F: TTGAAGCCTCTCGTTGACCC
	R: GTGGGATACAGCCAAACCCA
CYP3A4	F: CTTCATCCAATGGACTGCATAAAT
	R: TCCCAAGTATAACACTCTACACAGACAA
CYP3A7	F: AAACTTGGCCGTGGAAACCT
	R: CAGCATAGGCTGTTGACAGTC
EpCAM	F: AGAACCTACGGATCATCATTGAACTAA
	R: CGCGTTGTGATCTCCTTCTG
LGR5	F: CTCCCAGGTCTGGTGTGTTG
	R: GAGGTCTAGGTAGGAGGTGAAG
KRT19	F: ACCAAGTTTGAGACGGAACAG
	R: CCCTCAGCGTACTGATTTCCT
SOX9	F: GTACCCGCACTTGCACAAC

	R: TCTCGCTCTCGTTCAGAAGTC
GAPDH	F: AGAAGGCTGGGGCTCATTTG
	R: AGGGGCCATCCACAGTCTTC

CD133: prominin 1; EpCAM: epithelial cell adhesion molecule; LGR5: leucine-rich repeat-containing G-protein coupled receptor 5; KRT19: type 1 cytoskeletal 19 KRT19: type 1 cytoskeletal 19 (or keratin 19); SOX9: SRY-box transcription factor 9; GAPDH: glyceraldehyde 3-phosphate dehydrogenase; F: forward; R: reverse.

Table S2 List of primary antibodies used.

Primary antibody	Cat. #	Dilution factor
Albumin	ab207327, Abcam	1:200
EpCAM	ab71916, Abcam	1:100
HNF4α	ab41898, Abcam	1:200
KRT19	61029, Progen Biotechnik	1:100
LGR5	EPR3065Y, Genetech	1:100
MRP2	ab15603, Abcam	1:200
ZO-1	402200, Thermo Fisher Scientific	1:200
PE Anti-ASGPR 1	563655, BD Pharmingen™	1:50
FITC Anti-A1AT	A80-122F, Bethyl Laboratories, Inc.	1:200

EpCAM: epithelial cell adhesion molecule; HNF4α: hepatocyte nuclear factor 4-alpha; KRT19: type 1 cytoskeletal 19 KRT19: type 1 cytoskeletal 19 (or keratin 19); LGR5: leucine-rich repeat-containing G-protein coupled receptor 5; MRP2: multidrug resistance-associated protein 2; ZO-1: zona occludens protein 1; ASGPR: asialoglycoprotein receptor 1; A1AT:

alpha-1-antitrypsin.

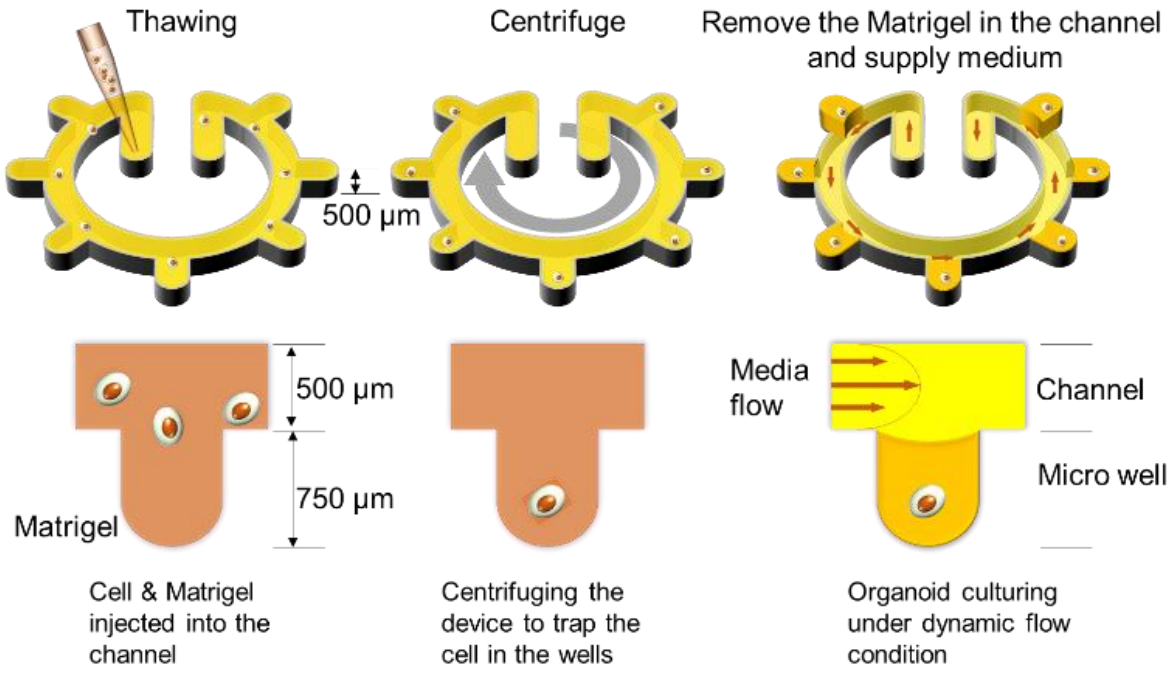

Figure S1. Overview of the seeding and trapping of human hepatic bipotent stem cells on the microphysiological system.

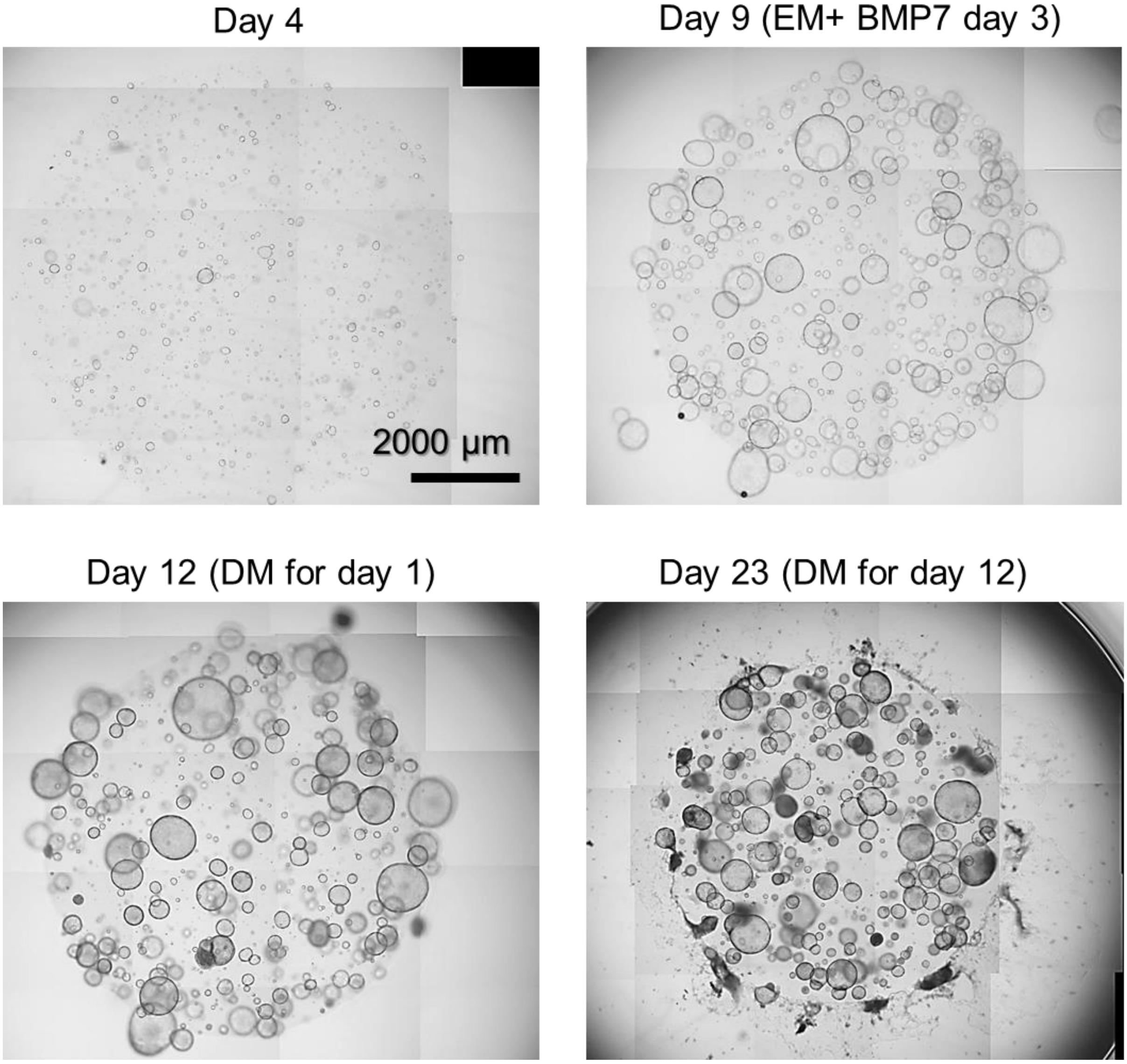

Figure S2. Variation in the growth of tissue-derived organoids in 3D Matrigel droplet cultures.

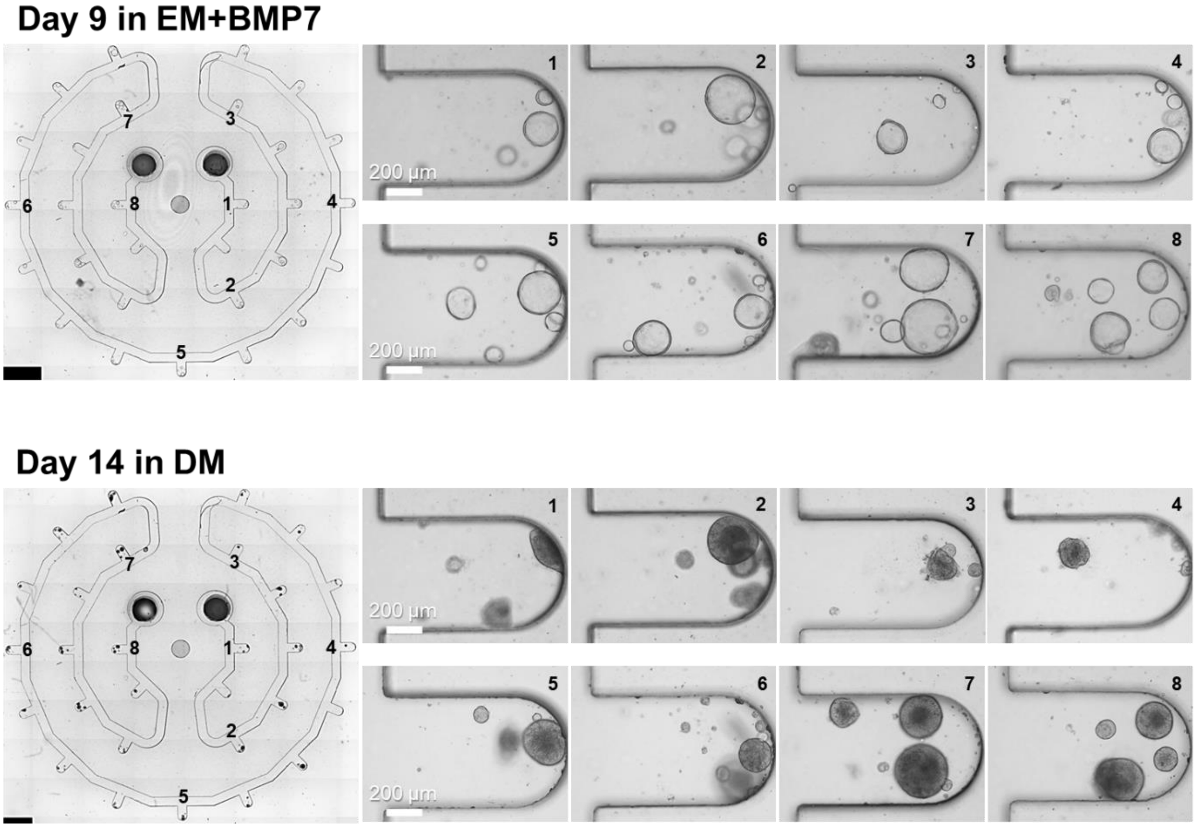

Figure S3. Representative examples of the morphology of human liver organoids in each of the indicated microwells.

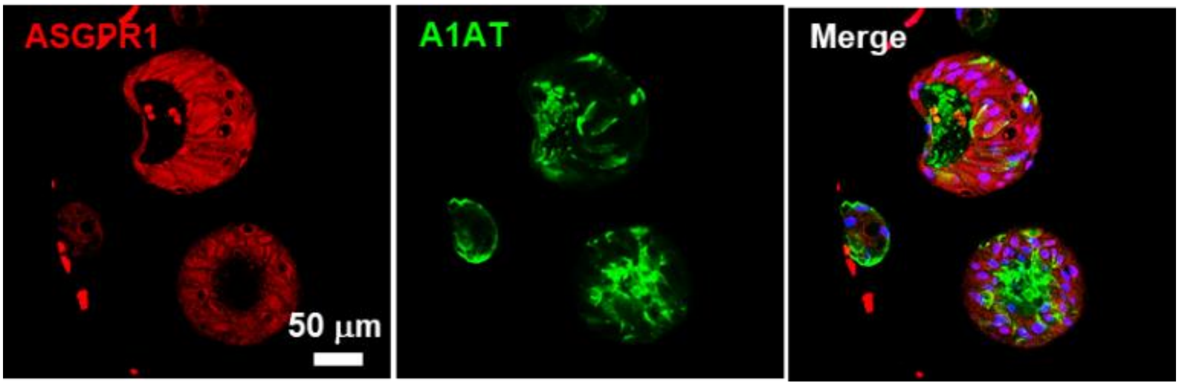

Figure S4. Whole-mount immunofluorescence staining of ASGPR1 and A1AT after hepatic differentiation under flow condition, followed by tissue clearing.
